# Current Evidence for the Prevention of Endophthalmitis in Anti-VEGF Intravitreal Injections

**DOI:** 10.1155/2018/8567912

**Published:** 2018-07-24

**Authors:** P. ET. Lau, K. S. Jenkins, C. J. Layton

**Affiliations:** ^1^College of Medicine and Dentistry, James Cook University, Townsville, Australia; ^2^Greenslopes Clinical School, Faculty of Medicine, University of Queensland, Brisbane, Australia

## Abstract

Intravitreal injection of a therapeutic substance is the most common procedure performed in ophthalmology. It has a low incidence of serious complications but is associated with a small chance of endophthalmitis. Although the rate of endophthalmitis is between 0.019% and 0.09%, the associated visual morbidity is often devastating. Procedural changes have evolved over the years to improve patient comfort and reduce injection-related injury and infection. Despite the availability of published evidence, there remains considerable variations and lack of consensus in practical clinical settings. In addition, emerging literature concerning the use of speculums, the use of prophylactic topical antibiotics, and the setting of injections continues to impact the ophthalmologist's injection practice. This article provides an up to date assessment of various aspects of the procedure such as the setting, ventilation, type of anaesthetic, and control of sterility during the procedure; including discussions on performing bilateral eye same-day injections and the use of antibiotics.

## 1. Introduction

Intravitreal injection (IVI) is the most commonly performed ophthalmic procedure and is likely the most commonly performed surgical procedure in medicine. The use of the intravitreal route of administration of anti-VEGF agents to treat diabetic macular oedema, exudative age-related macular degeneration, and retinal vasoocclusive disease has proven to be a legitimate medical breakthrough, and their use has grown dramatically [1]. The most feared complication of intravitreal injection is endophthalmitis, which is associated with a poor visual prognosis even with prompt diagnosis and treatment with intravitreal antibiotics or vitrectomy, and visual loss is common [2]. The incidence of endophthalmitis varies amongst studies. Various multicentre randomized controlled trials of anti-VEGF administration have reported incidences between 0.019% and 0.09%; however, these figures are in prospective trial conditions [3, 4]. Nonetheless, a systematic review of 20 large retrospective case series supported these figures, with an estimated endophthalmitis rate of 0.028% (1/3,544 injections) [5]. As a reference range for this review, the range between 0.019% and 0.09%, is used.

Anti-VEGF injections are used in a growing population of patients, and as treatment is maintained in existing patients, the number of treatment events continues to grow. This is advantageous to the patient, but more treatment events yield more chances for complications and more focus is required in planning to reduce the outcome of endophthalmitis. Despite the current availability of evidence, there are considerable variations and a lack of consensus and inconsistencies in clinical practice. Given the importance of the topic, the scientific literature relating to various aspects of the procedure is frequently updated, with recent controversies particularly surrounding the prophylactic use of topical antibiotics, same-day bilateral injections, and the setting of injections. This paper summarises the latest developments, reviews established evidence-based aspects of endophthalmitis prophylaxis in intravitreal injections, presents selected general scientific data of direct application to the ophthalmologist when optimising their injection technique, and highlights areas where further clinical research is required.

## 2. Topical Irrigation/Antisepsis

### 2.1. Povidone Iodine

Ophthalmic or “half strength” povidone-iodine is routinely used in ophthalmic surgery due to its broad spectrum antimicrobial activity, low incidence of microorganism resistance, cost-effectiveness, and wide availability [6]. As endophthalmitis is hypothesized to occur due to inoculation or ingress of microorganisms into the globe during injection, sterilizing of the ocular surface is of paramount importance and the central evidence-based recommendation of any injection protocol.

The omission of topical antiseptic is associated with significantly higher rates of endophthalmitis. In a study reported by DRCR.net, 3123 eyes received 28,786 intravitreous injections, usually with povidone-iodine preparation. However, a total of 13 injections in 2 participants were administered without antiseptic and both participants developed endophthalmitis in 1 eye each. This was 15% risk of endophthalmitis per injection. 100% of the at risk subjects develop endophthalmitis during the short duration of the treatments [7].

To date, the technique, concentration, and contact time of povidone-iodine still remains a matter of study. For example, a technique of a 10 ml flush of 5% povidone-iodine through the conjunctival fornix provided greater reduction of bacteria than a 2-3 drop application of 5% povidone-iodine [8] and a 30 sec exposure time to 5% povidone-iodine is associated with a significantly greater reduction in bacterial load than 15 secs [9].

While it is considered a necessity to use povidone-iodine or alternative antiseptic as an aseptic measure, repeated exposure of ocular surface does seem to lead to increasing reports of dry eye symptoms. Therefore, there is a need for health professionals to be aware of these changes and to manage these symptoms and risks [10].

### 2.2. Chlorhexidine

Aqueous chlorhexidine 0.1% is reported to be a good alternative to povidone-iodine for antisepsis in patients with significant postprocedure pain for intravitreal injection. In a pain survey scale of 0–10, patient discomfort is reported to be lower (3/10) using chlorhexidine compared with 8/10 using 5% povidone-iodine [11]. In addition, the efficacy of chlorhexidine is similar to povidone-iodine when investigating ocular bacterial count after antisepsis [12, 13]. In a multicentre retrospective case series using aqueous chlorhexidine, the endophthalmitis rate was 0.0074%, which compares favourably with povidone-iodine [13].

Although chlorhexidine compares well with povidone-iodine preparation and is widely used, there are reports of resistance in methicillin-resistant *Staphylococcus aureus* and fungi. On the other hand, there are no similar reports of resistance with povidone-iodine use [14].

### 2.3. Saline/Balanced Salt Solution

Although saline irrigation is ineffective in reducing bacteria count and may even increase bacterial count coming from the fornices, postinjection pain is often reported by patients as a result of the irritant effect of povidone-iodine or other antiseptics [12, 15]. It shows saline irrigation postinjection does decrease discomfort. In an attempt to decrease patient discomfort and to minimize epithelial toxicity, practitioners do irrigate ocular surfaces after intravitreal injection regardless of whether chlorhexidine or povidone-iodine is used as the antiseptic agent. Unfortunately, there is no evidence on the safety or otherwise of this practice.

Topical antiseptic is therefore critical to use prior to intravitreal injection, providing relative sterility in the immediate injection environment. The most widely preferred antiseptic is 5% povidone-iodine with contact time of at least 30 secs. Despite adequate topical anaesthesia, povidone-iodine can cause significant postoperative ocular surface irritation; however, no data on the safety of subsequent irrigation of the ocular surface with saline are available.

## 3. Setting of Procedure: Office Setting versus Operating Theatre

The modern intravitreal injection procedure is a modified posterior chamber paracentesis manoeuvre of traditional vitreoretinal surgery, and therefore many centres initiated early anti-VEGF treatments in the operating room environment. More recently, surveys assessing the settings for delivering intravitreal injections have shown a preference for an office or clean room environment, including a study from the UK which reported 83% of intravitreal injections were performed in a dedicated clean room with sterile preparation [16] and in Canada, where a majority of intravitreal injections were performed in the office setting [17]. Whether the setting of administration of intravitreal therapy influences the incidence of endophthalmitis has been widely debated. Certainly, an operative setting can be associated with exceeding small incidences of endophthalmitis: in a 2017 retrospective multicentre study of 134,701 injections across Europe, an endophthalmitis rate of 0.0074% was achieved in operating theatres [18]. Tabendeh et al. conducted a retrospective review of a total of 11,710 intravenous injections, where 8,647 intravitreal injections were done in an office-based setting and 3,063 were performed in the operating room. 5 endophthalmitis cases (0.043%–0.035% in office-based procedures and 0.065% in operating room procedures) were with no significant difference in the rate of endophthalmitis between the groups [19]. These results are further supported by a meta-analysis which showed no safety benefits in the administration of intravitreal injection in either the outpatient clinic, surgery, or at another location [20]. There are, however, other reports that the operating theatre is safer than the office-based setting. Abell et al. reported that operating room procedures were associated with a 13-fold decreased risk of endophthalmitis compared office-based procedures. The usual quoted confounding bias in Abell's study was patients who had injections in theatre were privately insured, while those who have their injections in office were not, [21] however, it must be commented that other retrospective studies may well have admitted more complicated or higher risk patients for operating room procedures, creating a reverse bias more likely to favour the office-based setting.

The figures also highlight the principle difficulty in performing studies and interpreting the results of all papers concerning postinjection endophthalmitis: namely, due to its very low incidence, even very large treatment cohorts yield very small numbers of affected individuals. Prospective trials of sufficient power to assess interventions for effectiveness must often number in the tens of thousands to achieve statistically significant differences between groups, and depending on the cost or invasiveness of the intervention being assessed, even statistically significant differences may not be clinically relevant.

Given the evidence shows it is safe to give intravitreal therapy either in office setting or operating room, it seems more cost effective, convenient, and efficient to perform intravitreal injections in an office setting. Nonetheless, this is surprising as first principles would seem to indicate that the negative pressure environment of the operating theatre should be safer, and many surgeons understandably prefer to treat their patients in the operating theatre environment for this reason.

## 4. Ventilation

Part of the reason for the hypothesis that the operating theatre is a safer setting for intervitreal injection is the theoretical importance of air-borne particles in causing infection. The efficacy of ultraclean air in significantly reducing infection rates was first demonstrated in orthopaedic implant surgery [22].

One new study trialled the use of a mobile ultraclean unidirectional airflow screen (UDF) to reduce airborne particles in simulated intravitreal injections. In the study, the UDF mobile screen reduced the mean particle concentration (particles >0.3 microns) over the stimulated ocular surface by at least a factor of 436 (*p* < 0.05) and on the instrument table by a factor of at least 100 (*p* < 0.05), clinically translating into significantly reduced air contamination. It appears the mobile UDF screen may provide a theoretically safer procedural environment for intravitreal injections in the office setting, but there remains no evidence that this will translate into lower endophthalmitis rates [23].

Indeed the relationship between air changes per hour and incidence of postoperative endophthalmitis generally has not been established. The ESCRS conducted a study of postphacoemulsification endophthalmitis comparing minimal airflow, 20 air changes per hour, and ultraclean air systems using either horizontal or vertical laminar airflow systems with no clear results [24]. It is proposed a similar future study could be carried out for postintravitreal injection endophthalmitis.

## 5. Respiratory Droplet Reduction

Face masks minimize aerosolisation of orophrayngeal droplets and are used throughout surgical specialties to reduce infection. The most commonly cultured bacteria in injection-related endophthalmitis are *Staphylococcus* species followed by *Streptococcus,* [25] and since *Streptococcus* are normal commensal organisms of the upper respiratory and oral cavities but usually not the conjunctival membranes, it has been suggested that dispersion of oral flora from either the treating physician or the patient themselves is one mechanism of endophthalmitis [26].

In a meta-analysis of endophthalmitis postintravitreal injection, McCannal reported 31% were due to *Streptococcus* species, a figure 3 times higher than that in other surgical procedures [27]. A retrospective study to assess the effect of a 2 year “no talking” period during the injection procedure reported the stringent policy was associated with a decreased rate of postinjection endophthalmitis, and a reduction of *Streptococcus-*associated endophthalmitis from 0.015% to 0.002% [28]. In another study, the use of a face mask and avoidance of talking significantly decreased the dispersion of respiratory flora during intravitreal injections. However, this microbiological study of a simulated injection environment also reported that even without a face mask or silence, use of povidone-iodine was associated with the least bacterial growth, demonstrating once again the paramount importance of conjunctival antisepsis in the procedure [29]. These findings have been widely adopted: in the UK, for example, 82% of 125 ophthalmologists used sterile adhesive eye drapes to isolate patients' nasopharyngeal area and periocular region [30].

Based on these findings, it would appear prudent for all staff members and patients who are present in the room to wear a mask during procedure setup and performance and to be instructed to minimize talking. Although maintaining silence is preferred, these findings must be weighed against the clinical importance of patient instructions and reassurance whilst performing the procedure.

## 6. Use of Gloves

The literature would indicate there is a surprisingly wide variation in clinical practise regarding the use of gloves, sterile or nonsterile, during intravitreal injection. In a survey of 717 US-based retinal specialists, 51% reported using 1 pair of gloves, 46% reported no gloves, and 3% used 2 pairs of gloves during intravitreal injections [31], whilst in Israel, 75% of 72 retinal specialists reported wearing sterile gloves for intravitreal injections [32].

Consistent with modern-day medical practise of invasive procedures, it is the authors' opinion that the use of sterile gloves is generally recommended. However, the scientific basis of this perception is challenged by a study conducted by Bhavsar et al. in which the study protocol required only nonsterile gloves and the rate of endophthalmitis was low. It was argued that the primary requirement for endophthalmitis prophylaxis was a procedural needle which remained sterile and a prepared eye surface is prepared [4]. Similar findings are reported in another study where the rate of endophthalmitis was 0.057% per injection in a total of 14,895 injections administered without the use of sterile gloves [33].

There is therefore insufficient data supporting the role of sterile gloves in reducing risk of postintravitreal injection endophthalmitis. Therefore, a practitioner may argue against the necessity of sterile gloves in this procedure with some degree of evidence-based backing; however, in many jurisdictions their use is likely to be mandatory.

## 7. Hand Hygiene and Antisepsis

The practise of hand hygiene is a fundamental element of medical practice, and general hand hygiene practice is to perform hand antisepsis regardless of the intention to use gloves. Although there are no studies directly linking type of hand antisepsis with endophthalmitis, the ophthalmologist should be aware of the efficacy of different antisepsis solutions, particularly when planning office-based infrastructure for an intraocular procedure. There are 2 main types of antisepsis solutions: aqueous scrubs and alcohol rubs. The major aqueous scrubs can be further divided into povidone-iodine, chlorhexidine, and triclosan [34].

In a randomized control trial, studying the effects of conventional chlorhexidine, povidone-iodine, and alcohol hand rub, chlorhexidine and the alcohol rub provided better antiseptic effectiveness than povidone–iodine, [35] and in general, alcohol rubs have superior antimicrobial efficacy than aqueous scrubs [36, 37] with a rapid reduction of microbial count and bacterial regrowth to baseline taking more than 6 hours [38].

Nonetheless, a Cochrane review investigating all hand antisepsis approaches concluded there was no firm evidence that one type of hand antisepsis was better than another, although alcohol rubs with additional antiseptic ingredients may reduce colony-forming units, and all the evidence of hand antisepsis available were of low or very low quality [34]. In real-life clinical practise, it is observed that alcohol hand rub may be a more favourable choice for surgical staff due to its antimicrobial efficacy, speed, and comfort.

## 8. Eyelid Retraction and Speculums

Eyelids, eyelid glands, and eyelashes are sources of infection in intraocular surgery regardless of antiseptic solution used, and surgical instruments must avoid touching them where possible. An eye speculum is one way to avoid contamination of the procedural needle, and previous studies suggest it lowers endophthalmitis rates by preventing causative pathogens contaminating the needle or the injection field [39]. In the United States, 92% of retinal specialists who participated in a survey stated they routinely used an eyelid speculum [31]. In the VISION trial of pegaptanib, no speculum was used with some patients, and the endophthalmitis rate was high at 0.18% per injection, but much higher amongst those patients in whom the speculum was not used [40].

Alternative techniques in isolating eyelids and eyelashes may also be acceptable in achieving the aim of the lid speculum. In a retrospective case series of 78,009 intravitreal injections, a lid-splinting eyelid retraction technique was used and an endophthalmitis rate of 0.015% was reported, similar to that of a lid speculum [41]. The finding is interesting because usage of an eyelid speculum is often associated with discomfort: a recent prospective study reported a majority of patients prefer bimanual eyelid retraction in comparison to a metal lid speculum as it was associated with less pain and discomfort [42].

Nonetheless, most practitioners have limited experience with alternative methods of eyelid retraction and studies must be conducted in other centres to compare the safety benefit and consistency of alternative eyelid retraction techniques prior to any discontinuation of lid speculum use for intravitreal injections.

## 9. Prophylactic Antibiotics

Topical prophylactic antibiotics have wide applications in surgery, and their use was initially extended to intravitreal injections, as it was assumed that they would reduce the risk of postinjection endophthalmitis in the same way that topical fourth generation fluoroquinolones and intracameral antibiotics reduced endophthalmitis risk in phacoemulsification surgery.

There is currently no trial that shows prophylactic topical antibiotics reduce the risk of postinjection endophthalmitis; however, the mechanism of this lack of efficacy remains unknown. In a retrospective case-control study of 117,171 injections, prophylactic antibiotic use was not associated with a change in the endophthalmitis rate (0.049% with antibiotics; 0.032% without) [43]. A more surprising finding is that of two meta-analyses which conclude prophylactic antibiotics actually increase the risk of endophthalmitis. In the first, data from 276,774 injections were analysed: 39.45% with prophylactic antibiotics and 60.55% without. The analysis of this data showed the risk of endophthalmitis was actually 1.70 times higher (*p*=0.02) with antibiotics than without. In the second, a pooled analysis of 639,391 anti-VEGF intravitreal injections, the incidence of endophthalmitis with topical antibiotic prophylaxis was three times with antibiotic use (0.09% versus 0.03%) [44].

The mechanism has been thought to be due to increasing the ratio of antibiotic-resistant surface bacteria and the overall pattern of resistance of periocular flora [20, 45] or the repeated use of fluoroquinolones having a detrimental effect on ocular surface health. Fluoroquinolone drops certainly result in increased resistance rates: in one study the conjunctival flora showed an 87.5% antibiotic resistance rate after 4 days of treatment [46].

Whilst this theory certainly explains a trend towards more challenging cases of endophthalmitis, the mechanism of a greater chance of bacterial ingress into the globe during the procedure remains unclear to the authors. If routine antibiotic use is prospectively shown to be associated with an increased endophthalmitis rate (as opposed to severity), alternative explanations could include the use of any postoperative drop or irrigation leading to ocular surface disturbance or increased tear film movement over the operative site, or increased handling of the eye by the patient leading to a higher chance or postoperative wound leak, thereby increasing the chance of postoperative ingress of infective organisms.

Unsurprisingly, recent evidence indicates an abandonment of prophylactic topical antibiotic use in the practice of some practitioners, but this has not been based on the results of prospective data. Given equivalent trials and their success in phacoemulsification surgery, such a trial would not be unreasonable, and currently the use of prophylactic topical antibiotics is still the usual practice for a significant percentage of practitioners. Regardless, it seems uncontroversial that, given the frequency of injections, the practitioner must assume that preoperative and postoperative courses of antibiotics will cause substantial ocular surface antibiotic resistance.

## 10. Anaesthesia

Patients often experience pain during intravitreal injections and the use of local anaesthetics aim to minimize discomfort and avoid pain-induced rapid or uncontrolled movements of the eye [47]. Any protocol designed to minimize endophthalmitis must both sterilise the surgical field but also maintain patient comfort and compliance whilst minimising patient movement, and both the effectiveness and sterility of the anaesthesic method are obviously central to this effort.

Currently, there is lack of consensus of which anaesthetic techniques are the best option for either maintaining sterility or patient compliance for intravitreal injections. A recent systematic review evaluating the effectiveness of different local anaesthetic techniques for intravitreal injections included local eye drop anaesthetics (tetracaine, proparacaine, and cocaine), lidocaine gel, lidocaine pledget, subconjunctival anaesthesia, and peribulbar block. It concluded patient discomfort was mild regardless of technique; however, it stated subconjunctival injection may be an option for highly sensitive patients [48], and this is supported by a survey of patients' preference of topical anaesthesia, which revealed 88% of the patients preferred subconjunctival anaesthesia over 12% of topical anaesthesia for ongoing intravitreal injections [49].

Andrade et al. reported a subconjunctival injection of 2% lidocaine was more effective for intravitreal injections compared to lidocaine gel or proparacaine drops [50]. Lidocaine itself has mild antiseptic properties, and Tustin et al. found no cases of culture positive endophthalmitis in a retrospective series of 6,583 injections using subconjunctival lidocaine versus 8 cases in a series of 8,189 cases using other methods (*p*=0.03), implying the significant difference could be due to anaesthetic's aseptic nature. The actual mechanism could, of course, be multifactorial and is likely to include some combination of the extended contact time of povidone-iodine, some unknown diluting or separating effect of the conjunctival bleb, the mild antiseptic effect of the agent itself, and improved intraoperative and postoperative patient compliance.

It should be noted that topical lidocaine gel has also been reported as a barrier to topical antiseptics reaching the ocular surface in *in vitro* models [51]. When applied prior to povidone-iodine, lidocaine gel application has been associated with an increased rate of postinjection endophthalmitis [52], but when povidone-iodine was applied first, subsequent lidocaine gel did not increase the rate of endophthalmitis in a retrospective series of 8802 injections [53]. It would therefore appear prudent to apply copious antiseptic agent to the surgical site as early as possible in the procedure and certainly before gel or subconjunctival anaesthesia is administered.

## 11. Bilateral Intravitreal Injection

In many ophthalmologists' practices, same-day bilateral intraocular interventions have historically been studiously avoided due to the risks of bilateral blindness from an unknown systematic cause on the day. Nonetheless, the incidence of bilateral diabetic macular oedema and choroidal neovascularisation has meant that it has become increasingly common for patients to request bilateral same-day treatment, and many clinicians are beginning to offer this service. Taking an independent risk of endophthalmitis at the upper end of the range, at 0.09% or 1/1,111, the risk of bilateral blindness of two completely independent procedures should be 1 in 1.2 million injections. Unfortunately, bilateral intravitreal injections are not independent procedures: even if separate packs, drapes, and lot numbers are used, the room, proceduralists, and patient are the same. The 1/1,111 risk is based on a unilateral injection and an already uncomfortable patient undergoing and recovering from a second procedure will have binocular visual impact and bilateral visual discomfort and is therefore unlikely to have such low risks. An untrained attendant, a ventilation problem on the day of the procedure, a problem in the patient's home, or an iatrogenic infection caused by an incipient URTI in any participant all make the independent probability calculation essentially worthless to the practioner's decision of whether to offer the service.

In a study conducted in a veteran hospital, 660 patients received bilateral same-day injection. Both eyes were prepared and injected sequentially without reuse of instruments or medications. No serious ocular complications including bilateral infectious or noninfectious endophthalmitis or retinal detachment were noted when standard aseptic protocol was observed [54]. In another study, Ruao et al. reported no culture-proven endophthalmitis after bilateral injection and a lower rate of acute intraocular inflammation relative to a unilateral treatment group (0.062% versus 0.152%) [55]. A lower infection rate in bilateral injections was also reported in a study by Ruao [55], where severe intraocular inflammation rates of 0.274% in 8172 unilateral injections compared favourably to 0.062% of 1612 bilateral injections. All these studies suggest that bilateral same-day injections are relatively safe clinical decision to make or recommend. However, the studies do have some caveats, especially the high rates of intraocular inflammation in unilaterally treated patients in Ruao's study and the low power of the other studies compared with the numerous single-eye studies. In addition, in the absence of an unknown physiological mechanism, the apparently lower level of inflammation/infection in the bilaterally treated patients can only reasonably be interpreted as either patient selection bias towards healthier patients for the bilateral option or increased care on the part of the procedural team when performing the bilateral procedures. This complicates interpretation of these studies substantially.

The clinical outcome of a bilateral case of endophthalmitis is perhaps easier for the clinician to interpret. Tabatabelli [56] reported 2 cases of bilateral endophthalmitis in patients with diabetic macular oedema, with final vision of light perception in one patient and 20/400 in the other. A second report (unpublished data) presented at the 2016 Australian and New Zealand Society of Retinal Specialists Meeting reported a patient who presented with unilateral low grade vitreous cells 2 days following bilateral intravitreal injections and underwent a sterile vitreous tap and injection of intravitreal antibiotics. 2 days later, the same patient presented with frank endophthalmitis in the other eye, subsequently losing the vision but fortunately retaining the vision in the first. There has also been an interesting report of bilateral reactivation of herpes simplex keratitis following bilateral intravitreal injections [57].

Nonetheless, although same-day bilateral treatment has not been studied in randomized or large controlled trials, the practice is thought to have become more common as it is generally well tolerated and preferred by patients and administrators [58]. Despite retrospective results to the contrary, there is no credible evidence that bilateral injections reduce the risk of injection associated endophthalmitis. If undertaken, it is imperative that intravitreal treatment of each eye be considered as a separate, sterile procedure, conducted with new lot numbers and without reusing instruments or medications to avoid increasing the risk of bilateral endophthalmitis.

## 12. Conclusion

This review aimed at examining factors that can be used to reduce or prevent post-intravitreal injection-related endophthalmitis. The authors believe there is evidence to recommend (in order of strength of evidence): povidone-iodine antisepsis (aqueous chlorhexidine where this is not possible), eyelid retraction with speculum, prevention of droplet spread via masks, adhesive drapes and reduced talking, and subconjunctival anaesthetic with lidocaine base agent. In addition to this, good sterile procedure and compliance with local policies including hand hygiene, use of gloves, staff training, and ensuring the chosen operative setting is optimised to the surgeon's satisfaction, whether a theatre or clean room is chosen. Bilateral same-day injections may be safe and can be implemented in clinical practice; however, the clinical risks of one patient complication may outweigh the convenience benefits in many practitioners' minds. The omission of prophylactic topical antibiotics seems justified by the existing literature; however, prospective trials are lacking. Mobile airflow screens, alternative eyelid retraction methods, and the question of whether postprocedure saline irrigation is beneficial or detrimental are all important developments to be explored in the future.

It should be stressed that in all of these cases, evidence quality is low and the difficulties discussed in studying a complication that occurs only once in every 1 to 4 thousand interventions means a clinicians' surgical skills, general training, clinical perspective, and experience continues to have a substantial role in preventing adverse events in this, the most commonly performed surgical procedure in ophthalmology.

## References

[1] Lanzetta P., Mitchell P., Wolf S., Veritti D. (2013). Different antivascular endothelial growth factor treatments and regimens and their outcomes in neovascular age-related macular degeneration: a literature review. *British Journal of Ophthalmology*.

[2] Sachdeva M. M., Moshiri A., Leder H. A., Scott A. W. (2016). Endophthalmitis following intravitreal injection of anti-VEGF agents: long-term outcomes and the identification of unusual micro-organisms. *Journal of Ophthalmic Inflammation and Infection*.

[3] Mason J. O., White M. F., Feist R. M. (2008). Incidence of acute onset endophthalmitis following intravitreal bevacizumab (Avastin) injection. *Retina*.

[4] Bhavsar A. R., Googe J. M., Stockdale C. R. (2009). Risk of endophthalmitis after intravitreal drug injection when topical antibiotics are not required: the diabetic retinopathy clinical research network laser-ranibizumab-triamcinolone clinical trials. *Archives of Ophthalmology*.

[5] Merani R., Hunyor A. P. (2015). Endophthalmitis following intravitreal anti-vascular endothelial growth factor (VEGF) injection: a comprehensive review. *International Journal of Retina and Vitreous*.

[6] Grzybowski A., Kanclerz P., Myers W. G. (2018). The use of povidone-iodine in ophthalmology. *Current Opinion in Ophthalmology*.

[7] Bhavsar A. R., Glassman A. R., Stockdale C. R., Jampol L. M. (2016). Elimination of Topical Antibiotics for Intravitreous Injections and the Importance of Using Povidone-Iodine: Update From the Diabetic Retinopathy Clinical Research Network. *JAMA Ophthalmology*.

[8] Safar A., Dellimore M. C. (2007). The effect of povidone-iodine flush versus drops on conjunctival colonization before intravitreal injections. *International Ophthalmology*.

[9] Friedman D. A., Mason J. O., Emond T., McGwin G. (2013). Povidone-iodine contact time and lid speculum use during intravitreal injection. *Retina*.

[10] Saedon H., Nosek J., Phillips J., Narendran N., Yang Y. C. (2017). Ocular surface effects of repeated application of povidone-iodine in patients receiving frequent intravitreal injections. *Cutaneous and Ocular Toxicology*.

[11] Oakley C. L., Vote B. J. (2016). Aqueous chlorhexidine (0.1%) is an effective alternative to povidone-iodine for intravitreal injection prophylaxis. *Acta Ophthalmologica*.

[12] Oakley C., Allen P., Hooshmand J., Vote B. J. T. (2017). Pain and antisepsis after ocular administration of povidone-iodine versus chlorhexidine. *Retina*.

[13] Merani R., McPherson Z. E., Luckie A. P. (2016). Aqueous chlorhexidine for intravitreal injection antisepsis: a case series and review of the literature. *Ophthalmology*.

[14] Grzybowski A., Brona P. (2017). Povidone-iodine is still a premium antiseptic measure in ocular surgery. *Acta Ophthalmologica*.

[15] Boes D. A., Lindquist T. D., Fritsche T. R., Kalina R. E. (1992). Effects of povidone-iodine chemical preparation and saline irrigation on the perilimbal flora. *Ophthalmology*.

[16] Samia-Aly E., Cassels-Brown A., Morris D. S., Stancliffe R., Somner J. E. (2015). A survey of UK practice patterns in the delivery of intravitreal injections. *Ophthalmic and Physiological Optics*.

[17] Xing L., Dorrepaal S. J., Gale J. (2014). Survey of intravitreal injection techniques and treatment protocols among retina specialists in Canada. *Canadian Journal of Ophthalmology*.

[18] Freiberg F. J., Brynskov T., Munk M. R. (2017). Low endophthalmitis rates after intravitreal anti-vascular endothelial growth factor injections in an operation room: a retrospective multicenter study. *Retina*.

[19] Tabandeh H., Boscia F., Sborgia A. (2014). Endophthalmitis associated with intravitreal injections: office-based setting and operating room setting. *Retina*.

[20] Bande M. F., Mansilla R., Pata M. P. (2017). Intravitreal injections of anti-VEGF agents and antibiotic prophylaxis for endophthalmitis: a systematic review and meta-analysis. *Scientific Reports*.

[21] Abell R. G., Kerr N. M., Allen P., Vote B. J. (2012). Intravitreal injections: is there benefit for a theatre setting?. *British Journal of Ophthalmology*.

[22] Dharan S., Pittet D. (2002). Environmental controls in operating theatres. *Journal of Hospital Infection*.

[23] Lapid-Gortzak R., Traversari R., van der Linden J. W., Lesnik Oberstein S. Y., Lapid O., Schlingemann R. O. (2017). Mobile ultra-clean unidirectional airflow screen reduces air contamination in a simulated setting for intra-vitreal injection. *International Ophthalmology*.

[24] Endophthalmitis Study Group (2007). Prophylaxis of postoperative endophthalmitis following cataract surgery, results of the ESCRS multicenter study and identification of risk factors. *Journal of Cataract & Refractive Surgery*.

[25] Yu C. Q., Ta C. N. (2014). Prevention and treatment of injection-related endophthalmitis. *Graefe’s Archive for Clinical and Experimental Ophthalmology*.

[26] Chen E., Lin M. Y., Cox J., Brown D. M. (2011). Endophthalmitis after intravitreal injection: the importance of viridans streptococci. *Retina*.

[27] McCannel C. A. (2011). Meta-analysis of endophthalmitis after intravitreal injection of anti-vascular endothelial growth factor agents: causative organisms and possible prevention strategies. *Retina*.

[28] Garg S. J., Dollin M., Hsu J., Storey P., Vander J. F. (2015). Effect of a strict ‘No-Talking’ policy during intravitreal injection on post-injection endophthalmitis. *Ophthalmic Surgery, Lasers & Imaging Retina*.

[29] Doshi R. R., Leng T., Fung A. E. (2012). Reducing oral flora contamination of intravitreal injections with face mask or silence. *Retina*.

[30] Huang K., Sultan M. B., Zhou D., Tressler C. S., Mo J. (2016). Practice patterns of ophthalmologists administering intravitreal injections in Europe: a longitudinal survey. *Clinical Ophthalmology*.

[31] Green-Simms A. E., Ekdawi N. S., Bakri S. J. (2011). Survey of intravitreal injection techniques among retinal specialists in the United States. *American Journal of Ophthalmology*.

[32] Segal O., Segal-Trivitz Y., Nemet A. Y., Geffen N., Nesher R., Mimouni M. (2016). Survey of intravitreal injection techniques among retina specialists in Israel. *Clinical Ophthalmology*.

[33] Cheung C. S., Wong A. W., Lui A., Kertes P. J., Devenyi R. G., Lam W. C. (2012). Incidence of endophthalmitis and use of antibiotic prophylaxis after intravitreal injections. *Ophthalmology*.

[34] Tanner J., Dumville J. C., Norman G., Fortnam M. (2016). Surgical hand antisepsis to reduce surgical site infection. *Cochrane Database of Systematic Reviews*.

[35] Tuuli M. G., Liu J., Stout M. J. (2016). A randomized trial comparing skin antiseptic agents at cesarean delivery. *New England Journal of Medicine*.

[36] Shen N. J., Pan S. C., Sheng W. H. (2015). Comparative antimicrobial efficacy of alcohol-based hand rub and conventional surgical scrub in a medical center. *Journal of Microbiology, Immunology and Infection*.

[37] Lai K. W., Foo T. L., Low W., Naidu G. (2012). Surgical hand antisepsis-a pilot study comparing povidone-iodine hand scrub and alcohol-based chlorhexidine gluconate hand rub. *Annals of the Academy of Medicine, Singapore*.

[38] Rotter M. L., Kampf G., Suchomel M., Kundi M. (2007). Population kinetics of the skin flora on gloved hands following surgical hand disinfection with 3 propanol-based hand rubs: a prospective, randomized, double-blind trial. *Infection Control & Hospital Epidemiology*.

[39] Fineman M. S., Hsu J., Spirn M. J., Kaiser R. S. (2013). Bimanual assisted eyelid retraction technique for intravitreal injections. *Retina*.

[40] Gragoudas E. S., Adamis A. P., Cunningham E. T., Feinsod M., Guyer D. R. (2004). Pegaptanib for neovascular age-related macular degeneration. *New England Journal of Medicine*.

[41] Munro M., Williams G. R., Ells A. (2017). Lid splinting eyelid retraction technique: a minimised sterile approach for intravitreal injections. *British Journal of Ophthalmology*.

[42] Rahimy E., Fineman M. S., Regillo C. D. (2015). Speculum versus bimanual lid retraction during intravitreal injection. *Ophthalmology*.

[43] Storey P., Dollin M., Pitcher J. (2014). The role of topical antibiotic prophylaxis to prevent endophthalmitis after intravitreal injection. *Ophthalmology*.

[44] Reibaldi M., Pulvirenti A., Avitabile T. (2018). Pooled estimates of incidence of endophthalmitis after intravitreal injection of anti-vascular endothelial growth factor agents with and without topical antibiotic prophylaxis. *Retina*.

[45] Hsu J., Gerstenblith A. T., Garg S. J., Vander J. F. (2014). Conjunctival flora antibiotic resistance patterns after serial intravitreal injections without postinjection topical antibiotics. *American Journal of Ophthalmology*.

[46] Milder E., Vander J., Shah C., Garg S. (2012). Changes in antibiotic resistance patterns of conjunctival flora due to repeated use of topical antibiotics after intravitreal injection. *Ophthalmology*.

[47] Gambrell J., Schaal S. (2012). Topical anesthesia for intravitreal injection. *Expert Opinion on Drug Delivery*.

[48] Shiroma H. F., Takaschima A. K. K., Farah M. E. (2017). Patient pain during intravitreal injections under topical anesthesia: a systematic review. *International Journal of Retina and Vitreous*.

[49] Cohen S. M., Billiris-Findlay K., Eichenbaum D. A., Pautler S. E. (2014). Topical lidocaine gel with and without subconjunctival lidocaine injection for intravitreal injection: a within-patient study. *Ophthalmic Surgery, Lasers & Imaging Retina*.

[50] Andrade G. C., Carvalho A. C. (2015). Comparison of 3 different anesthetic approaches for intravitreal injections: a prospective randomized trial. *Arquivos Brasileiros de Oftalmologia*.

[51] Tustin A., Kim S. J., Chomsky A., Hubbard G. B., Sheng J. (2014). Antibacterial properties of 2% lidocaine and reduced rate of endophthalmitis after intravitreal injection. *Retina*.

[52] Inman Z. D., Anderson N. G. (2011). Incidence of endophthalmitis after intravitreal injection of antivascular endothelial growth factor medications using topical lidocaine gel anesthesia. *Retina*.

[53] Lad E. M., Maltenfort M. G., Leng T. (2012). Effect of lidocaine gel anesthesia on endophthalmitis rates following intravitreal injection. *Ophthalmic Surgery, Lasers, and Imaging*.

[54] Chao D. L., Gregori N. Z., Khandji J., Goldhardt R. (2014). Safety of bilateral intravitreal injections delivered in a teaching institution. *Expert Opinion on Drug Delivery*.

[55] Ruao M., Andreu-Fenoll M., Dolz-Marco R., Gallego-Pinazo R. (2017). Safety of bilateral same-day intravitreal injections of anti-vascular endothelial growth factor agents. *Clinical Ophthalmology*.

[56] Tabatabaii A., Ahmadraji A., Khodabande A., Mansouri M. (2013). Acute bilateral endophthalmitis following bilateral intravitreal bevacizumab (avastin) injection. *Middle East African Journal of Ophthalmology*.

[57] Derham A. M., Chen E., Bunya V. Y., O’Malley R. E. (2017). Bilateral herpetic keratitis after bilateral intravitreal bevacizumab for exudative macular degeneration. *Cornea*.

[58] Giocanti-Auregan A., Tadayoni R., Grenet T. (2016). Estimation of the need for bilateral intravitreal anti-VEGF injections in clinical practice. *BMC Ophthalmology*.

